# Detecting and enumerating soil-transmitted helminth eggs in soil: New method development and results from field testing in Kenya and Bangladesh

**DOI:** 10.1371/journal.pntd.0005522

**Published:** 2017-04-05

**Authors:** Lauren Steinbaum, Laura H. Kwong, Ayse Ercumen, Makeda S. Negash, Amira J. Lovely, Sammy M. Njenga, Alexandria B. Boehm, Amy J. Pickering, Kara L. Nelson

**Affiliations:** 1 Civil and Environmental Engineering, Stanford University, Stanford, California, United States of America; 2 School of Public Health, University of California, Berkeley, Berkeley, California, United States of America; 3 Integrative Biology, University of California, Berkeley, Berkeley, California, United States of America; 4 Eastern and Southern Africa Center of International Parasite Control, Kenya Medical Research Institute (KEMRI), Nairobi, Kenya; 5 Civil and Environmental Engineering, University of California, Berkeley, Berkeley, California, United States of America; Facultad de Medicina, Universidad Nacional Autonoma de Mexico, UNITED STATES

## Abstract

Globally, about 1.5 billion people are infected with at least one species of soil-transmitted helminth (STH). Soil is a critical environmental reservoir of STH, yet there is no standard method for detecting STH eggs in soil. We developed a field method for enumerating STH eggs in soil and tested the method in Bangladesh and Kenya. The US Environmental Protection Agency (EPA) method for enumerating *Ascaris* eggs in biosolids was modified through a series of recovery efficiency experiments; we seeded soil samples with a known number of *Ascaris suum* eggs and assessed the effect of protocol modifications on egg recovery. We found the use of 1% 7X as a surfactant compared to 0.1% Tween 80 significantly improved recovery efficiency (two-sided t-test, t = 5.03, p = 0.007) while other protocol modifications—including different agitation and flotation methods—did not have a significant impact. Soil texture affected the egg recovery efficiency; sandy samples resulted in higher recovery compared to loamy samples processed using the same method (two-sided t-test, t = 2.56, p = 0.083). We documented a recovery efficiency of 73% for the final improved method using loamy soil in the lab. To field test the improved method, we processed soil samples from 100 households in Bangladesh and 100 households in Kenya from June to November 2015. The prevalence of any STH (*Ascaris*, *Trichuris* or hookworm) egg in soil was 78% in Bangladesh and 37% in Kenya. The median concentration of STH eggs in soil in positive samples was 0.59 eggs/g dry soil in Bangladesh and 0.15 eggs/g dry soil in Kenya. The prevalence of STH eggs in soil was significantly higher in Bangladesh than Kenya (chi-square, χ^2^ = 34.39, p < 0.001) as was the concentration (Mann-Whitney, z = 7.10, p < 0.001). This new method allows for detecting STH eggs in soil in low-resource settings and could be used for standardizing soil STH detection globally.

## Author summary

Intestinal worm infections are common in populations living in tropical, low-income countries. People primarily become infected when they consume intestinal worm eggs from contaminated water, hands, and food. Intestinal worm eggs are transmitted from infected people and spread through the environment, particularly via soil. There is no standard laboratory method for counting intestinal worm eggs in soil, which is a major barrier to comprehensive research on the transmission of infection. We tested different laboratory protocol steps to extract soil-transmitted helminth eggs, which is one type of intestinal worm, from soil and propose a new, fast, and efficient field method. We tested the method in Kenya and Bangladesh and found that soil contamination with helminth eggs was prevalent in both study areas. We propose that environmental contamination be included in discussions about intestinal worm transmission, control, and elimination, especially in areas with low infection prevalence. The method we propose will help researchers assess soil contamination, which can be used to examine the effectiveness of intestinal worm transmission control measures.

## Introduction

Almost one quarter of the world’s population is infected with at least one species of soil-transmitted helminth (STH) [1]. South Asia, Southeast Asia, and Sub-Saharan Africa are the regions with the highest prevalence [1]. *Ascaris* and *Trichuris* infection is spread via an environmentally-mediated fecal-oral transmission route, through ingestion of a larvated egg that has incubated in soil. Hookworm infection is spread by larvae, hatched from eggs after incubation in the soil, penetrating the skin. One hookworm species, *Ancylostoma duodenale*, can also be transmitted by ingestion of a larvae [2]. Although soil is the main environmental reservoir of STH eggs, there is no standard method for enumerating STH eggs in soil. In contrast, there is a relative abundance of data on global STH infection prevalence as measured by detection of eggs in stool, in part, because there are standard stool analysis methods that are relatively fast and appropriate for resource-constrained settings [3].

A standard method for counting STH eggs in soil would be valuable for examining soil as a key step in the transmission pathway, and to compare field studies that have estimated the prevalence of STH in soil. Previous field methods have focused on three main steps: sieving, flotation, and microscopy. The number and size of sieves used varies. In general, a large sieve is used to remove large soil particles and a small sieve is used to retain STH eggs. Flotation methods can vary based on the flotation solution and the flotation time. Many flotation solutions have been used, including magnesium sulfate [4–6], zinc sulfate [7–9], sodium nitrate [10], sugar [11–16], and salt [17]. Magnesium sulfate has been recommended by the US Environmental Protection Agency (US EPA) for detection of *Ascaris* in wastewater and biosolid samples [6]. Zinc sulfate has been commonly used for flotation of parasite eggs in stool samples and is very effective, but zinc is toxic to aquatic life and requires safe disposal. Sugar is inexpensive and easily accessible, but it can distort STH eggs and make microscopic identification difficult [18]. Sugar solutions also attract flies and are susceptible to microbial growth, requiring the addition of an antimicrobial substance such as formaldehyde [19]. Salt is inexpensive and easily accessible, but the specific gravity reaches a maximum at ~1.2. Although this specific gravity should be high enough to recover *Ascaris*, *Trichuris*, and hookworm spp., it may not be high enough to recover heavier parasite eggs, such as *Taenia* spp. [20].

Differences in the egg flotation step are also attributed to the use of passive versus centrifugal floatation methods. Passive flotation can be useful in low-resource settings because it does not require access to a centrifuge, but it may not be feasible for a large number of samples because it requires more time. Methods that use centrifugal flotation report varied centrifuge speed, number of flotation steps, and flotation times. Additionally, some methods rely on adherence to a coverslip while centrifuging, which can reduce processing time, but it is not known how this affects the recovery efficiency [21]. As reviewed by Collender et al., there is limited research on the impact of different steps in the protocol on the recovery efficiency of the method [21], which may have contributed to the lack of a standard protocol. Additionally, many methods with published recovery efficiencies use *Toxocara* eggs [21] as a test organism instead of *Ascaris* eggs, which are slightly smaller and denser than *Toxocara* [20].

Another challenge is that there are few references that provide guidance on correctly identifying STH eggs in soil through microscopy. Researchers need substantial experience reviewing slides from soil samples before they are able to distinguish the different STH egg types from the debris and animal parasite eggs common in soil samples. The most widely used resource for STH egg identification is a bench guide created by the World Health Organization for identification of parasites in clinical samples [22]. There is also a guide for analysis and identification of helminths eggs in wastewater [18], but there is no similar resource for identification of parasites in soil samples. Laboratory technicians familiar with identification of STH eggs in stool samples are not qualified to analyze soil samples without additional training, as soil contains different life stages of STH eggs, non-STH eggs, and debris.

Molecular methods, such as DNA extraction followed by quantitative polymerase chain reaction (qPCR), offer the potential to reduce human error in egg identification compared to microscopy. Molecular methods are under development for detection of STH in stool and biosolids [23,24]. Additionally, two recent studies used molecular methods for detection of hookworm species and *Ascaris lumbricoides* in soil [25,26]. Molecular methods should be also be created and tested for detection of *Ascaris* and *Trichuris* in soil. Potential complications with developing and employing molecular methods in the field for enumerating STH eggs in soil include: cost, accessibility of reagents and equipment, inhibition of assays from humic and fulvic acids present in soil [27], and ability to distinguish viable and non-viable eggs [28]. These issues must be resolved to make molecular detection of STH in soil a feasible method for low-resource settings.

The goal of this study was to develop and evaluate a field method based on direct microscopy for quantifying STH eggs in soil. After reviewing previous field methods and their limitations, we tested the impact of different protocol steps on the egg recovery efficiency in the laboratory to inform an improved protocol. The aim was to shorten the processing time and make the method easier to implement in remote field laboratories, without negatively impacting recovery efficiency. We assessed the feasibility of using the new method in a low-resource setting by field testing it in Kenya and Bangladesh. Using data from our field tests, we also compared the prevalence and concentration of STH eggs in soil in Kenya and Bangladesh.

## Methods

### Ethics statement

The study procedures were approved by the Stanford Institutional Review Board (Protocol Numbers 23310 [Kenya] and 25863 [Bangladesh]), the Kenya Medical Research Institute (KEMRI) Ethical Review Committee (SSC Number 2271), and the International Center for Diarrheal Diseases Research, Bangladesh (icddr,b) Ethical Review Committee (PR-11063).

### Recovery efficiency experiments

We based our initial method [29] on the US EPA method for detecting and enumerating *Ascaris* eggs in wastewater, sludge, and compost [6], which has not been previously validated for use with soil. The US EPA method employs a series of sample concentration and flotation steps. After the laboratory processing, *Ascaris* eggs are counted using microscopy. The main benefits of this method are that it is a standard method for biosolids in the US and the recovery efficiency is high. For example, a recent study found the efficiency of the method for recovering helminth eggs from composted feces and sugarcane husk was 71.6% [30]. The main challenge of the US EPA method is that it is time consuming; the protocol takes approximately three days. Therefore, we reduced the time and number of settling steps to reduce the overall processing time while varying several protocol elements such as sieve size and surfactant and flotation solutions to enhance recovery efficiency.

We performed experiments at Stanford University to determine the *Ascaris* egg recovery efficiency of protocol variations by analyzing seeded samples. We collected organic loam and sand from two different locations at Stanford University to use in the experiments. *Ascaris suum* eggs were purchased from Excelsior Sentinel, Inc. (Trumansburg, NY). Eggs were collected from intestinal contents of infected pigs and preserved in 0.1 N sulfuric acid. *Ascaris suum* eggs have been used in other laboratory experiments as a proxy for *Ascaris lumbricoides* eggs because they have a lower health risk to humans, they are easily procured, and they are morphologically identical to *Ascaris lumbricoides* [24,31,32]. Eggs were stored at 4°C prior to use. To seed soil samples, we counted a 1 mL aliquot of eggs suspended in distilled water (mean = 931 eggs, standard deviation = 128 eggs) under a microscope and rinsed them into a 50-mL centrifuge tube containing 15 g of soil. The initial concentration of seeded STH eggs in soil was approximately 62 eggs/g wet soil. Seeded soil samples were left to sit for one day at room temperature prior to laboratory processing to allow the eggs to percolate into the soil and adhere to soil particles. Each recovery efficiency experiment was conducted on three independent samples (experimental triplicates).

### Method protocol testing

We focused our experiments on three different aspects of the protocol and a total of eight processing steps associated with these to assess their impact on egg recovery: (1) egg detachment (choice of surfactant, stir-plate mixing), (2) concentrating the sample (settling time, settling volume, sieve size), and (3) egg flotation (flotation time, specific gravity of the flotation solution). We also tested different soil textures, loam and sand, to determine the effect of soil type on the recovery efficiency.

#### Egg detachment

To evaluate the effect of egg detachment on recovery efficiency, we tested two surfactants (Table 1, Column A) and the addition of a stir-plate mixing step for increased agitation (Table 1, Column B). The surfactants were 0.1% Tween 80 and 1% 7X. Tween 80 is a nonionic, viscous surfactant and 7X is an anionic, non-viscous surfactant. 1% 7X was shown to more efficiently recover *Ascaris* from biosolids and hands in hand rinse samples than 0.1% Tween 80 [33,34]. We also tested the use of a mixing step where we mixed the sample in a 1000 mL beaker with surfactant up to 600 mL for 10 minutes using a stir plate and magnetic stir bar. While this is expected to enhance the detachment of eggs by increasing agitation, there is also some evidence that mixing with a magnetic stir bar can reduce the viability of *Ascaris* eggs, and that this effect is greater for mammilated eggs than for decorticated eggs [32]. However, we only focused on egg recovery and did not test the viability of *Ascaris* in our recovery efficiency experiments.

**Table 1:** Recovery efficiency for methods to extract STH eggs from soil.

	A	B	C	D	E	F	G	H	
	Surfactant	Stir-plate mixing step [Y/N]	Settling time [min]	Settling volume [mL]	Sieve size [mesh size]	Flotation step [min]	Zinc sulfate specific gravity	Soil texture	Mean recovery efficiency [% (SD)]
1	0.1% Tween 80	Y	120	600	400	1 x 10	1.20	loam	37.2 (3.3)
2	1% 7x	Y	120	600	400	1 x 10	1.20	loam	53.0 (3.0)
3	1% 7x	Y	120	600	400	1 x 10	1.20	sand	67.6 (7.5)
4	1% 7x	Y	120	600	500	1 x 10	1.20	loam	58.5 (6.1)
5	1% 7x	Y	120	600	500	2 x 5	1.20	loam	68.0 (5.4)
6	1% 7x	N	120	600	500	2 x 5	1.20	loam	59.9 (6.0)
7	1% 7x	N	30	150	500	2 x 5	1.20	loam	62.5 (0.3)
8	1% 7x	N	30	150	500	1 x 10	1.20	loam	44.5 (5.6)
9	1% 7x	N	30	150	500	1 x 10	1.25	loam	65.6 (6.3)
10	1% 7x	N	30	150	500	2 x 5	1.25	loam	72.7 (3.9)

SD = standard deviation of experimental triplicates

#### Concentrating the samples

We tested several settling volumes (Table 1, Column D), settling times (Table 1, Column C), and sieve sizes (Table 1, Column E) to evaluate the effect on recovery efficiency of different steps that contribute to concentrating the eggs. We tested settling volumes of 600 and 150 mL, and settling times of 2 hours and 30 minutes. The settling velocity of STH eggs in wastewater is 0.58 m/hr for *Ascaris* and 0.32 m/hr for *Trichuris* [35], suggesting that the settling time in a standard 1000-mL beaker for a settling volume of 600 mL is approximately 8 minutes for *Ascaris* and 15 minutes for *Trichuris*, and the settling time for 150 mL is 2 minutes for *Ascaris* and 4 minutes for *Trichuris*. Finally, we tested a 400-mesh sieve (37 μm) and 500-mesh sieve (25 μm) for the final sieving step. *Trichuris trichiura* eggs range in size from approximately 57 x 26 μm to 78 x 30 μm [36]; therefore, small eggs can fit through the openings of a 400-mesh sieve on their short axis. Although we did not test *Trichuris* egg recovery in these experiments, we evaluated whether using a 500-mesh sieve would clutter the Sedgwick-Rafter cell with fine soil particles and make microscopic enumeration difficult.

#### Egg flotation

To evaluate the effect of egg flotation on recovery efficiency, we tested different flotation solution specific gravities (Table 1, Column G) and flotation times (Table 1, Column F). The specific gravity of *Ascaris suum* is 1.13 [20], so we tested 1.20 and 1.25 specific gravity zinc sulfate as the flotation solutions. We also examined two flotation procedures, one 10-minute step and two 5-minute steps.

### Improved protocol used for field validation

Based on the results from our recovery efficiency experiments, we developed an improved protocol and field-tested the method in Bangladesh and Kenya. We added a 15 g aliquot of soil to a 50 mL centrifuge tube to process soil samples for enumeration of STH eggs with the improved method. Then, we added surfactant, 1% 7X (MP Biomedicals, Santa Ana, CA), to each sample, bringing the volume up to the 35 mL line, and vigorously shook the samples by hand for two minutes. We rinsed the sides and cap of the tube with 1% 7X, added 1% 7X to the 45 mL line on the centrifuge tube, and left the samples to soak overnight. The next morning, we hand shook each sample for one minute, vortexed for 15 seconds, and poured through a stainless steel size 50-mesh sieve (300 μm, H&C Sieving Systems, Columbia, MD). We rinsed the sample through the sieve with 1% 7X and rinsed the bottom of the sieve with 1% 7X to capture any eggs stuck to the sieve. The settling volume was around 150 mL. We left the samples to settle for 30 minutes and then vacuum aspirated the supernatant. We poured the remaining sample into two 50-mL centrifuge tubes, filled the tubes to the 40 mL line with 1% 7X, and centrifuged (Sorvall Legend XT, Thermo Scientific, Waltham, MA) at 1000 x g for 10 minutes. We gently poured off the supernatant without disturbing the soil pellet, added 5 mL of zinc sulfate solution (ZnSO_4_ heptahydrate, 1.25 specific gravity) as flotation solution, vortexed for 30 seconds, and added additional zinc sulfate solution up to the 40 mL line. We centrifuged at 1000 x g for 5 minutes and then poured the supernatant through a fine stainless steel 500-mesh sieve (25 μm, H&C Sieving Systems, Columbia, MD). We rinsed the contents of the sieve into a clean 50 mL centrifuge tube (CELLTREAT, Shirley, MA) using distilled water. Then, we repeated this flotation step a second time using a clean sieve. We centrifuged the solution at 1000 x g for 5 minutes to settle the helminth eggs. We removed the supernatant using a clean 25 mL serological pipette until only 1 mL of solution remained. We transferred the final solution to a Sedgwick Rafter slide (1 mL cell volume, Wildco, Yulee, FL) using a pipettor (1000 μL fixed volume pipette, Cole Parmer, Vernon Hills, IL).

We examined slides using a microscope (M10 series, Swift, Schertz, TX) under 10x magnification to count *Ascaris*, *Trichuris* and hookworm eggs. We returned samples that contained any STH eggs back to their centrifuge tubes by rinsing the slide with distilled water and added 4 mL of 0.1 N sulfuric acid. We incubated these tubes at 28°C for 28 days. We reexamined these samples after incubation to determine egg viability by counting the number of larvated eggs that remained. Larvated eggs were considered viable whereas fertilized but non-larvated eggs were considered non-viable because they did not develop during the incubation period.

To determine moisture content, we dried an aliquot of each soil sample. Moisture content can vary widely based on local conditions, so it is necessary to report concentrations in terms of mass of dry soil. A 15 g aliquot of soil (wet weight) was placed on foil and oven dried overnight for at least 16 hours at 110°C in a gravity convection oven. Samples cooled for 10 minutes on a countertop before weighing to determine dry weight.

#### Soil texture characterization

**Fig 1:**
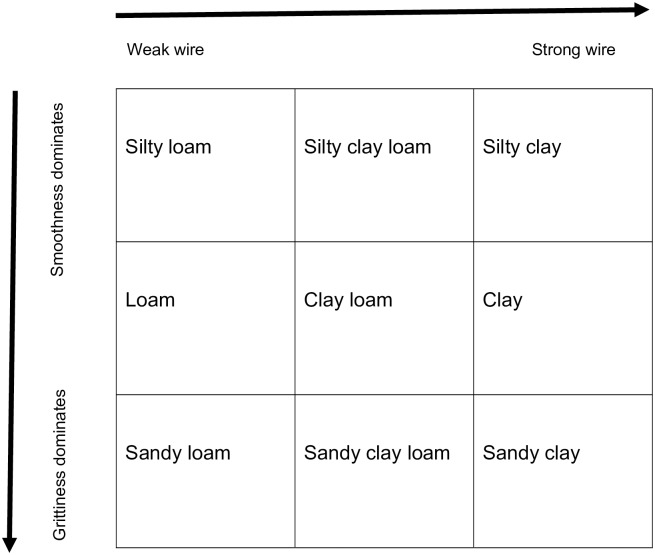
Determination of soil texture classification.

We assessed the soil texture of our samples because soil texture may influence STH egg viability and recovery efficiency. We performed soil texture characterization on aliquots of oven-dried soil rather than fresh soil samples, to reduce the risk of pathogen exposure. After determining the dry weight of soil from the oven-dried sample, we added water to the sample until the soil was just saturated but not glistening. We mixed the soil and water well and formed it into a small, thin wire [37]. Laboratory staff determined the clay content by whether the wire was strong, medium, or weak. Then, laboratory staff rubbed the soil between their fingers to determine the sand and silt content of the soil. Soil with high sand content is gritty and soil with high silt content is very smooth. We then determined the soil texture using our observations of the clay and sand content (Fig 1), based on the USDA soil texture triangle and flow chart for determining soil texture by feel [38].

#### Egg identification

**Fig 2:**
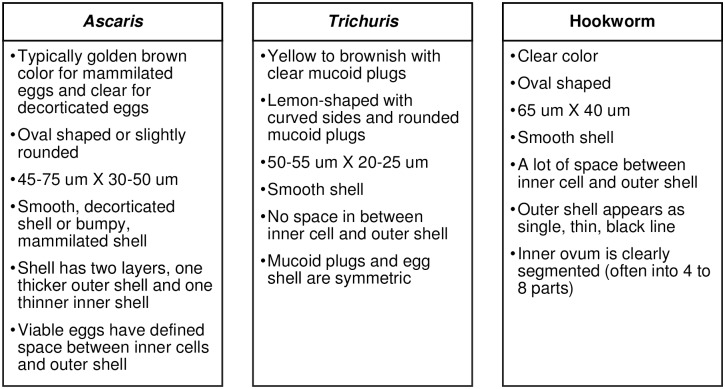
Characteristics of STH eggs.

We identified *Ascaris*, *Trichuris* and hookworm eggs based on the list of visual characteristics we developed (Fig 2) [22].

We also developed characteristics to distinguish *Trichuris* eggs from those of *Capillaria*, which look similar, but are unlikely to infect humans and are not of interest for quantifying the STH egg burden in soil. Although there are different *Capillaria* species from different animals, we summarized the main characteristics of the genus as a whole to help differentiate between *Capillaria* and *Trichuris*. *Capillaria* has a yellowish or light brown outer shell. The eggs are barrel-shaped with flat sides. In contrast to *Trichuris*, the shell is bumpy and pitted instead of being smooth. Also, the mucoid plugs can be asymmetric, unlike for *Trichuris* eggs. Instead of having rounded, protruding mucoid plugs, *Capillaria* can have flat or smaller mucoid plugs. Also, *Capillaria* eggs often have space between the inner cell and the outer shell [39].

### Field assessment of method in Kenya and Bangladesh

We field tested the improved protocol in Bangladesh from June to August 2015, during the rainy season, and in Kenya from August to November 2015, during the dry season and the beginning of the short rainy season. Field staff obtained written consent from all study participants on a prior visit, as well as oral consent on the day of soil collection. We collected soil samples from 100 rural households in Kakamega in western Kenya and 100 rural households in Mymensingh, Tangail, and Kishoreganj districts in central Bangladesh. We selected households based on their proximity to our field laboratories and their participation in an ongoing intervention trial [40]. We collected soil samples at the primary house entrance, either directly adjacent to the doorway or within 2 meters of the doorway if there was no soil in front of the doorway. Field staff collected soil by scraping the entire surface layer within a 900 cm^2^ area using a clean metal spade and scooping the soil into a sterile Whirlpak bag (118 mL capacity, Nasco, Fort Atkinson, WI). We collected approximately 50 g (wet weight) of soil from each household. Field staff transported samples at room temperature to our field laboratory and stored them in a 4°C refrigerator before laboratory processing began. Field staff in Bangladesh followed the same sample collection protocol as in Kenya, except that samples were transported on ice to the field laboratory.

Laboratory staff processed all samples using the improved protocol. In Kenya, we processed 7% of samples (7 out of 100 total samples) with a laboratory replicate. We counted 44 samples both pre- and post- incubation to determine the percentage of viable eggs and to act as quality assurance and quality control. We also took photos of the first egg seen in a sample for each type of egg for additional review. To ensure consistency across countries, both laboratory teams shared and reviewed each other’s egg photos. In Bangladesh, we made a few adaptations to the lab protocol used in Kenya. First, we increased the settling time from 30 minutes to at least 1 hour. Second, we oven dried 5 grams of soil instead of 15 grams to determine the moisture content. Third, we incubated all samples immediately after processing, instead of counting eggs pre- and post-incubation, due to logistical constraints. Finally, we did not characterize the soil texture of any of the Bangladeshi samples. In Bangladesh, we processed 9% of the samples (9 out of 100 total samples) with a laboratory replicate to assess the variability of the method, and two lab technicians counted 17% (17 out of 100 total samples) of samples in duplicate to assess inter-counter variability for quality assurance and quality control.

### Statistical analysis

We calculated recovery efficiency by dividing the final egg count by the initial egg count. We analyzed the results of the recovery efficiency experiments using two-sided t-tests to compare the experiments that had just one variation in the protocol (settling time, soil texture, sieve size, flotation time and number of steps, stir-plate mixing, settling volume and time, flotation solution specific gravity); we compared recovery efficiencies from three experimental triplicates to another three experimental triplicates. We assessed the difference in the prevalence (the proportion of positive samples) of any STH in soil in Kenya and Bangladesh using a chi-square test and the difference in the total concentration of STH eggs per dry gram of soil using a Mann-Whitney test. Any p-value less than 0.05 was considered to be statistically significant. We analyzed the recovery efficiency experiment results using Excel 2013 and the field results using STATA version 13.

## Results

### Recovery efficiency experiments

**Fig 3:**
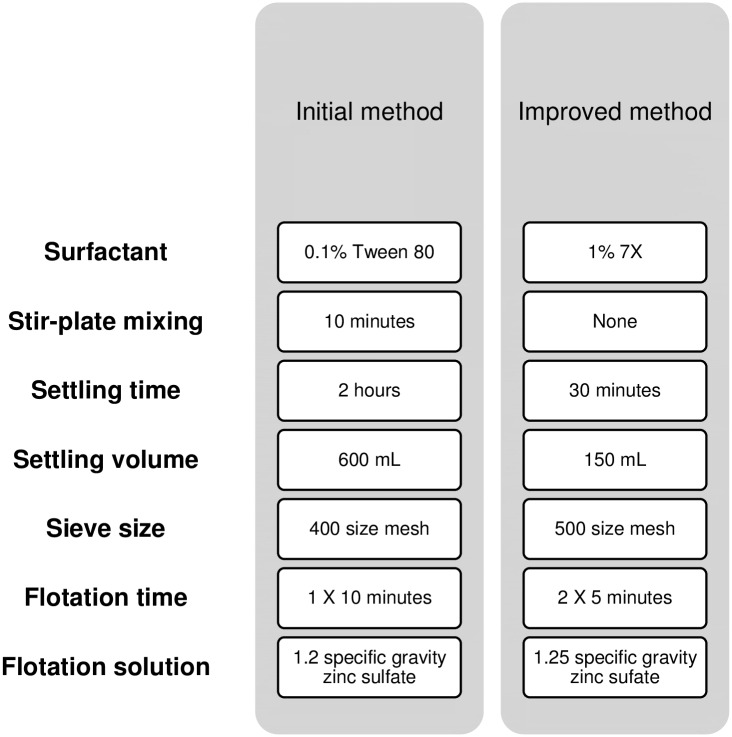
Comparison of initial and improved method for extracting STH eggs from soil.

We compared the use of 0.1% Tween 80 and 1% 7X in experiments 1 and 2; 1% 7X significantly improved *Ascaris* recovery by 16.2 percentage points over 0.1% Tween 80 (two-sided t-test, t = 5.03, p = 0.007) (Table 1: 2A *vs* 1A). This was the only change to the protocol that resulted in a statistically significant change in recovery efficiency; however, we made several other adaptions to the protocol based on the magnitude of the difference in recovery efficiencies and time savings. The impact of soil texture on recovery efficiency was assessed in experiments 2 and 3; the recovery efficiency was higher by 14.6 percentage points (two-sided t-test, t = 2.56, p = 0.083) when using sandy soil compared to loamy soil. The recovery efficiency using a 400-mesh and 500-mesh sieve was similar (experiments 2 and 4). In experiments 4 and 5, we compared using two 5-minute flotation steps and one 10-minute flotation step; using two 5-minute flotation steps resulted in an increase of 9.5 percentage points in recovery efficiency (two-sided t-test, t = 1.67, p = 0.171), so we adopted this flotation protocol. We compared the protocol without a stir-plate mixing step to the protocol with it (experiments 5 and 6), and although we found a slight decrease in recovery efficiency of 8.1 percentage points (two-sided t-test, t = 1.43, p = 0.226), we decided to remove this step to save time. Comparing experiments 6 and 7, there was no loss of recovery efficiency when we reduced the settling volume and time (two-sided t-test, t = 0.62, p = 0.601), so we adopted these changes. We compared 1.25 specific gravity with 1.2 specific gravity as a flotation solution and found a 10.2 percentage point increase in recovery efficiency when two 5-minute flotation steps were used (two-sided t-test, t = 3.65, p = 0.068) (experiments 7 and 10); we therefore decided to use a specific gravity of 1.25. The final, improved method had a significantly higher recovery efficiency (72.7%) than the initial method (37.2%) (two-sided t-test, t = 9.83, p < 0.001) (experiments 10 and 1). The differences between the initial and improved method are detailed in Fig 3.

### Field assessment of method in Kenya and Bangladesh

The prevalence of any STH eggs from our study area in Kenya was 37%. *Ascaris* was most common (22%), followed by *Trichuris* (21%) (Table 2). No hookworm eggs were found. Some *Ascaris* eggs were larvated (34.8%) when we isolated them from soil, but few *Trichuris* eggs were larvated (6.5%) prior to incubation. Most *Ascaris* eggs were viable (99.3%) and the majority of *Trichuris* eggs were viable post-incubation (71.6%) (Table 2). The median concentration in positive samples was 0.15 eggs/g dry soil (mean = 0.46 eggs/g dry soil) or 2 eggs/sample (mean = 6.1 eggs/sample). The soil texture of most soil samples in Kenya was either sandy loam (60%) or clay loam (31%). The mean moisture content of all samples in Kenya was 9.1%. The mean difference in egg counts before and after incubation was 3 eggs per sample.

**Table 2:** Prevalence, viability, and concentration of STH eggs in soil in Kenya and Bangladesh.

		Any STH	*Ascaris*	*Trichuris*	Hookworm
**Kenya**	**Prevalence of Eggs [%]**	37.0	22.0	21.0	0
	**Mean Percent of Eggs that were Viable [% (SD)]**	88.5 (26.1)	99.3 (2.6)	71.6 (42.0)	--
	**Range of Concentration of Eggs in Positive Samples [eggs/g dry soil]**	0.07–4.1	0.07–4.1	0.07–2.0	--
**Bangladesh**	**Prevalence of Eggs [%]**	78.0	67.0	36.0	0
	**Mean Percent of Eggs that were Viable [% (SD)]**	73.8 (36.8)	69.8 (40.5)	87.5 (27.0)	--
	**Range of Concentration of Eggs in Positive Samples [eggs/g dry soil]**	0.07–15.5	0.07–15.5	0.08–4.0	--

SD = standard deviation

The prevalence of any STH eggs in soil from our study area in Bangladesh was 78% (Table 2). Sixty-seven percent of samples contained *Ascaris* and 36% of samples contained *Trichuris*, while hookworm eggs were not detected (Table 2). *Ascaris* eggs had a similar viability as *Trichuris* eggs. (Table 2). The median concentration of positive STH eggs in soil was 0.59 eggs/g dry soil (mean = 1.6 eggs/g dry soil) or 8 eggs/sample (mean = 21 eggs/sample). The mean moisture content of all samples in Bangladesh was 19.1%. Soil samples from Bangladesh were significantly more likely to have STH eggs than soil samples from Kenya (chi-square, χ^2^ = 34.39, p < 0.001). Bangladeshi soil also had a significantly higher concentration of STH eggs as compared to Kenyan soil (Mann-Whitney, z = 7.10, p < 0.001). Slides counted twice by different enumerators had consistent counts, with a mean 9% difference in counts and an overall difference of about 1 egg per sample. The variation of egg counts in laboratory replicates was about 4 eggs/sample.

## Discussion

This paper presents an improved method for enumerating STH eggs in soil that is appropriate for use in resource-constrained settings. This field method is relatively fast; approximately 20 samples can be processed in a day and a half. In comparison, the original US EPA method takes at least 3 days to complete the full protocol on 10 samples. The method also has a higher recovery efficiency of 73% compared to previously published field methods. A recent review of previous methods for detecting STH eggs in environmental media demonstrated a median method recovery efficiency of 25% [21]. Our new protocol is comprehensive in that it includes STH egg enumeration, identification and viability determination, as well as soil moisture content measurement and soil texture classification.

Our recovery experiments in the lab identified one protocol step that affected recovery efficiency. We found that using 1% 7X instead of 0.1% Tween 80 significantly increased the egg recovery efficiency of the method. This result is consistent with a published method for enumerating *Ascaris* in hand rinse samples [33]. No other changes to the protocol were statistically significant; however, the recovery efficiency was significantly higher for the improved method that included several alterations than for the original method.

In our field tests, we found that the study area in Bangladesh had a higher prevalence and concentration of STH eggs in soil than the study area in Kenya. One factor that could affect the egg prevalence in soil is the infection prevalence of STH in the study area. STH infection is widely geographically variable [41–45], so it is important to examine the same study area for infection prevalence. An alternative explanation is that latrine access and sanitation infrastructure may be different in the two study areas. Also, flooding during the monsoon season in Bangladesh may spread waste from pit latrines and fecal sludge ponds to the surrounding areas. Other factors that are expected to influence the soil prevalence include sanitation behaviors and environmental conditions; these aspects are the focus of ongoing studies in both locations.

There are several limitations of our recovery efficiency experiments and field tests. We only tested one STH egg concentration (approximately 67 eggs/g wet soil) during the recovery efficiency experiments, and the concentration that was used to seed the samples was higher than what we typically found in soil in Kenya and Bangladesh. Recovery efficiency has been shown to change with the initial concentration of eggs in soil; one study found that recovery efficiency was inversely proportional to the egg concentration. Although they did not test concentrations as low as those that we would expect to see in naturally contaminated samples, this may indicate that the recovery efficiency of our method could be higher than 73% for samples with a low-concentration of STH eggs [17]. Another limitation is that we performed most of our recovery efficiency experiments with loamy soil, one experiment with sandy soil, and no experiments with clay soil. It is likely that the recovery efficiency would be highest in sand, followed by loam and then clay [21]. Thus, at a minimum it is important to note the soil texture when analyzing soil samples for the presence of STH eggs. Ideally, the impact of soil type on recovery efficiency should be measured in future studies, using samples from the actual field sites. Also, we had wide ranges in recovery efficiency between triplicates, limiting our power to detect statistically significant differences between protocol variations. Similarly, the ordering of our experiments may have affected the difference in recovery efficiency between steps because we did not test all potential combinations of the different steps. Finally, we did not test the recovery efficiency of the method for *Trichuris* or hookworm as, unlike *Ascaris*, these eggs cannot be easily procured in the United States; the recovery efficiency of the protocol may be different for these STH eggs than the value we report for *Ascaris*.

We did not detect hookworm in any of the soil samples. It is unclear whether hookworm was not present in our study areas or whether the protocol is not appropriate for detecting hookworm. As hookworm larvae hatch from eggs in the soil rather than in the human large intestine, we could expect to detect both eggs and larvae in the soil. Two studies that used sieving, centrifugation, and flotation steps similar to our protocol recovered hookworm eggs from seeded soil samples [4,7]. In one of these studies, 58% of the samples contained only larvae [4]. Our method should work in principle for recovering hookworm larvae if the samples are examined by microscopy immediately after processing, although it needs to be confirmed that larvae are not retained by the 50 mesh sieve (pore size of ~ 300 um) as infective filariform larvae are ~ 600 um in length. Also, hookworm eggs are fragile [21] and may be damaged by storage and processing. Thus, since the samples in Bangladesh were only enumerated after incubation, it was not expected to find hookworm eggs. Future work is needed to develop methods that are also effective for recovering hookworm eggs and larvae.

It should be noted that STH eggs from humans can be morphologically similar or identical to STH eggs from animals. For example, *Ascaris lumbricoides* eggs from humans are morphologically identical to *Ascaris suum* eggs from pigs [46]. Also, *Trichuris trichiura* eggs from humans appear like *Trichuris suis* eggs from pigs [47]. *Trichuris vulpis* eggs from dogs are larger than other *Trichuris* eggs, but there can be some overlap in the size ranges [48]. We identified STH eggs in our study based on a set of standard criteria (Fig 2). Although microscopy tends to be more feasible in low-resource settings than molecular methods, human error may occur and it is difficult to completely rule out accidental enumeration of morphologically similar eggs from animal sources. This is a particular concern for studies that seek to understand human fecal contamination in the environment or exposure to human STH eggs. More work should therefore be done to develop molecular methods for *Ascaris* and *Trichuris* in soil that can differentiate between eggs from human and animal hosts. In particular, inhibition from compounds in soil needs to be addressed before these assays can be deployed. Our cleaning and concentration method could be used in combination with molecular methods to reduce inhibition and increase the volume of processed soil before performing DNA extraction and PCR for detection of STH eggs.

The method presented here can be used to examine STH soil contamination to better understand STH transmission. It is relatively fast and efficient compared to other methods, making it more feasible for high-throughput processing in the field. A standard method for enumerating STH in soil will allow comparison of the prevalence and risk factors of soil contamination with STH across different settings, e.g. household sanitation practices (presence and type of latrine, management of child feces), community-level practices (presence of open drains, locations where fecal sludge is disposed or reused), and climatic and environmental effects. Soil contamination measurements can also be an effective tool for evaluating interventions aimed at reducing STH transmission.

## Supporting information

S1 Dataset *Ascaris* egg recovery efficiency experiment results. (XLSX)

S2 Dataset Kenya field assessment of method. (XLSX)

S3 Dataset Bangladesh field assessment of method. (XLSX)
